# Development and Validation of a Fast and Optimized Screening Method for Enhanced Production of Secondary Metabolites Using the Marine *Scopulariopsis brevicaulis* Strain LF580 Producing Anti-Cancer Active Scopularide A and B

**DOI:** 10.1371/journal.pone.0103320

**Published:** 2014-07-31

**Authors:** Annemarie Kramer, Linda Paun, Johannes F. Imhoff, Frank Kempken, Antje Labes

**Affiliations:** 1 Kiel Centre for Marine Natural Products at GEOMAR Helmholtz Centre for Ocean Research Kiel, Kiel, Germany; 2 Department of Genetics and Molecular Biology in Botany, Institute of Botany, Christians-Albrechts-University, Kiel, Germany; University of Florida, United States of America

## Abstract

Natural compounds from marine fungi are an excellent source for the discovery and development of new drug leads. The distinct activity profiles of the two cyclodepsipeptides scopularide A and B against cancer cell lines set their marine producer strain *Scopulariopsis brevicaulis* LF580 into the focus of the EU project MARINE FUNGI. One of the main goals was the development of a sustainable biotechnological production process for these compounds. The secondary metabolite production of strain LF580 was optimized by random mutagenesis employing UV radiation. For a fast and reliable detection of the intracellular secondary metabolite production level, a miniaturized bioactivity-independent screening method was developed, as the random mutagenesis yielded a large number of mutants to be analysed quantitatively and none of the existing hyphenated bioassay-dependent screening systems could be applied. The method includes decreased cultivation volume, a fast extraction procedure as well as an optimized LC-MS analysis. We show that deviation could be specifically reduced at each step of the process: The measuring deviation during the analysis could be minimized to 5% and technical deviation occurring in the downstream part to 10–15%. Biological variation during the cultivation process still has the major influence on the overall variation. However, the approach led to a 10-fold reduction of time and similar effects on costs and effort compared to standard reference screening methods. The method was applied to screen the UV-mutants library of *Scopulariopsis brevicaulis* LF580. For validation purposes, the occurring variations in the miniaturized scale were compared to those in the classical Erlenmeyer flask scale. This proof of concept was performed using the wild type strain and 23 randomly selected mutant strains. One specific mutant strain with an enhanced production behavior could be obtained.

## Introduction

Marine fungi are well known for their great potential to produce a broad range of secondary metabolites that benefit humankind, e.g. by their use as pharmaceuticals and cosmetics [1], [2]. Besides several antibiotic compounds, an increasing number of anti-cancer substances became known to be produced by marine fungi. Despite their proofed potential as drug producers, marine fungi still represent an underexplored source for new metabolites [3], [4]. Furthermore, the identification of new strains with the ability to produce substances using cultivation-based screening methods, is a task to be managed [5].

The fungus *Scopulariopsis brevicaulis* LF580 isolated from the inner tissue of the marine sponge *Tethya aurantium* collected in the Mediterranean Sea was shown to produce the two cyclodepsipeptides scopularide A and B. Both metabolites have specific activities against the pancreatic tumor cell lines (Colo357, Panc89) and the colon tumor cell line (HT29) [6], [7]. The development of these compounds as lead structures requires sufficient material supply. Hence, strain improvement by mutagenesis was applied in order to enhance the production of the two peptides. UV radiation is still the method of choice to gain a high number of mutants without using any targeted genetic tool [8]. However, the detection of changes within the production profile of these mutants is the challenging part in this process, since the number of mutant strains to be screened is enormous. In many mutagenesis screenings, the production of secreted metabolites or enzymes was measured in coupled assays using easy to read-out colorimetric assays (enzyme activities) or inhibition zone measurements in the case of antibiotic active compounds. Some secreted metabolites, e.g. sugars, small acids etc. can be detected directly by HPLC analysis of the culture broth [9]. For our research question, we needed a bioactivity-independent evaluation concept for intracellular secondary metabolites of fungal origin. A screening detecting such metabolites in a specific and quantitative way is a laborious and expensive task as it included, beside cultivation and specific chemical analyses, an additional extraction step. Therefore, cell wall disruption had to be included and was evaluated regarding the extraction grade. Thus, reducing procedural effort through the use of an optimized and fast screening system, based on small-scale cultivation and extraction, as well as an adapted analysis was required. Miniaturization can contribute to this, considering the given biological features of the strain of interest. Quantitative analysis concepts applicable for our specific task were not available, when we started our study. In addition, the fermentation of filamentous fungi below flask or tube scale is not common and information on the reliable production of secondary metabolites within smaller scales is generally scarce [10]. As secondary metabolism strongly depends on small environmental changes [11], downscaling may lead to changes in the metabolite spectrum in numerous cases. Small-scale cultivation is an analytical challenge anyway, as it yields a lower total amount of metabolite. Hence, the scale must allow production sufficient for detection [10].

Biological variation is an issue for all screening technologies available; however sufficient information on the influence of biological in contrast to methodological variation is scarce [9]. Hence, our main issue was the development of a robust and reliable screening system applicable generally for the search of secondary metabolites in fungi. Miniaturization of the entire screening process including all three parts - cultivation (upstream), extraction (downstream) and analysis - can generally enable the exploration of a wider range of process conditions and, therefore, the creation of robust and scalable bioprocesses will lower the need of time, costs and effort [12].

The mutagenesis experiments using *Scopulariopsis brevicaulis* LF580 are the respective application example. In contrast to other fungal producers, the wild type strain LF580 had a qualitatively stable production profile with a low number of major compounds. Besides the two scopularides, only paxilline was produced as a third major compound [6]. Therefore, strain LF580 can be considered a good candidate to perform random mutagenesis and to establish an optimized screening process for the detection of secondary metabolites produced by a non-model organism.

We present a method to contribute to the task of the quantification of secondary metabolites active against cancer cell lines that necessitates a cell disruption and LC-MS measurement in a high throughput approach. A special focus was set on the reliability of each part. To our knowledge, a method comprising all these aspects has not been published.

## Materials and Methods

### Strain

*Scopulariopsis brevicaulis* LF580 was taken from the strain collection of the Kiel Center for Marine Natural Products as cryoconserved material. It was originally isolated from the inner tissue of the marine sponge *Tethya aurantium* [6].

### Upstream

#### Conidia isolation and UV mutagenesis

**Figure 1:**
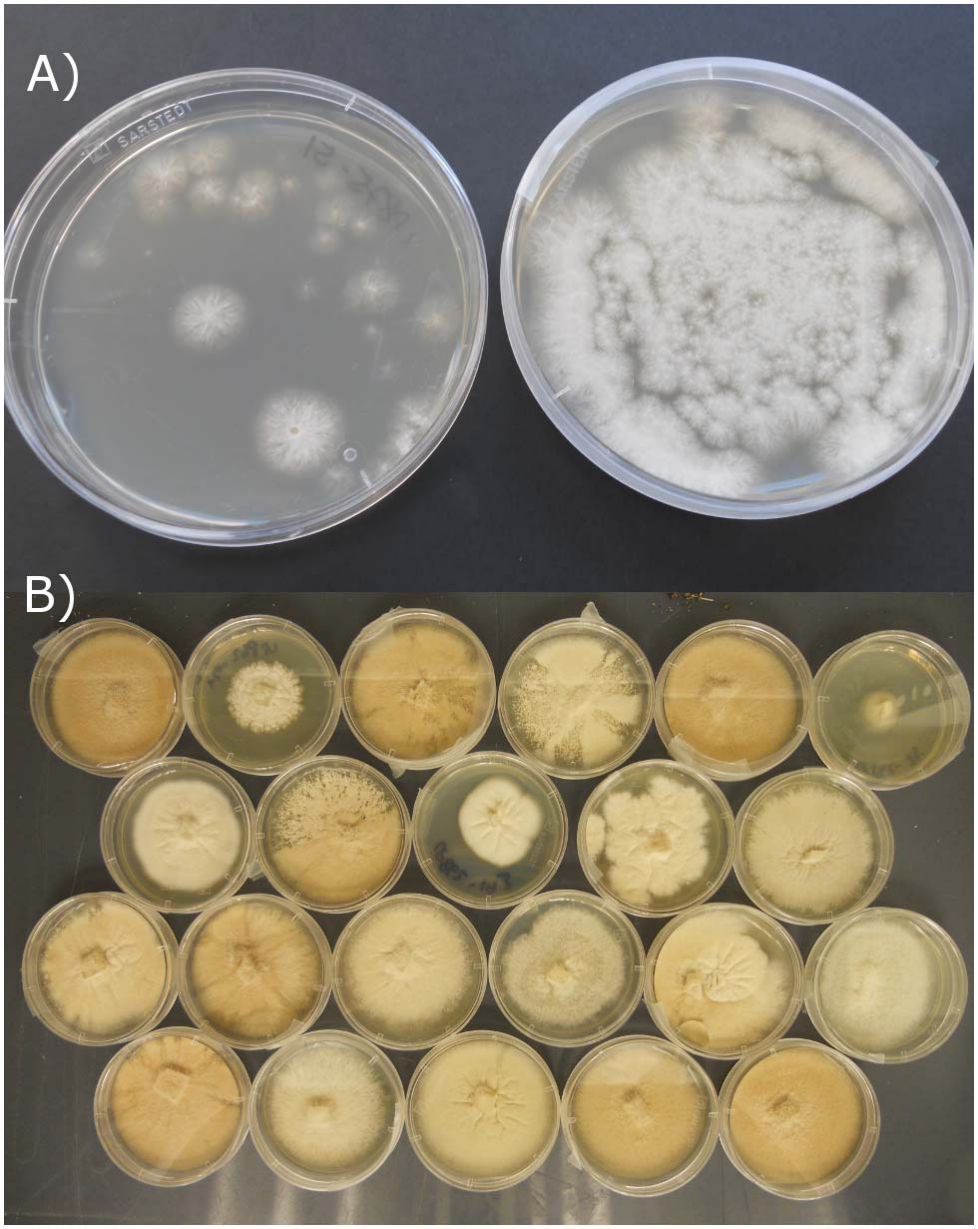
*Scopulariopsis brevicaulis* mutant strains on WSP30 petri dishes; a) Example of UV treated (left) and not treated (right) conidia growing on petri dishes: UV-radiation was performed using 312 nm wavelength for a time periods of 85 seconds and 13.742 W m^−2^ (85 s: 1.17 kJ m^−2^) radiation intensity. 1% (mean of 20 colonies) of the conidia were germinating; b) Colonies of different mutant strains growing on petri dishes upon UV mutagenesis.

Cultivation was carried out on solid WSP30 medium [13] (1% glucose×H_2_O, 0.5% soy peptone, 0.3% malt extract, 0.3% yeast extract, 3% NaCl, 2% agar) in an Erlenmeyer flask for one week at 25°C with a 16∶8 h day-night cycle, respectively. The conidia isolation was performed according to standard procedures: 75 mL of 0.9% NaCl solution with 0.1% Tween20 and 2 mM CaCl_2_×2 H_2_O was added to the Erlenmeyer flask and the conidia were dissolved from the agar by shaking. Conidia were filtered through four layers of mull and centrifuged at 4500 rpm (Beckman Coulter, Allegra x-22R) for 10 minutes. Isolated conidia were washed three times with 0.9% NaCl solution+2 mM CaCl_2_×2 H_2_O. 2000 conidia were plated on WSP30 solid medium for UV exposure. The survival rate was adjusted to less than 1%. UV-radiation was performed using 312 nm wavelength for different time periods. At 85 seconds exposure time and 13.742 W m^−2^ (85 s: 1.17 kJ m^−2^) radiation intensity, less than 1% (mean of 20 colonies) of the conidia were germinating. An example is shown in figure 1a. Due to the success of this radiation setting all further experiments were conducted using the same parameters.

#### Miniaturized cultivation set-up for UV-mutants

The colonies obtained after UV exposure were transferred to small petri dishes (60×15 mm, Sarstedt AG & Co., Nümbrecht) and incubated until a dense mycelium was built (Figure 1b). A small amount of conidia was transferred into 1 mL of liquid medium in 24-well plates (16×20 mm, Sarstedt AG & Co., Nümbrecht). This ensured the growth of a small mycelium which could be handled easily. The cultures were incubated for 4–5 days at 25°C, shaking at 100 rpm (Minitron, INFORS AG, Bottmingen). Approximately 100–200 mg of mycelia (wet weight) were obtained per well.

#### Reference cultivation

Cultivation was carried out in 300 mL Erlenmeyer flasks containing 100 mL WSP30 medium at 28°C, 120 rpm in the dark for 4 days. Agar pieces with 5 mm diameter of 4-day-old cultures were used as pre-cultures.

#### Influence of diurnal rhythm cultivation compared to constant dark cultivation

Cultures were compared regarding the influence of the light regime on the production profile of the target compounds scopularides A and B. Cultivation was carried out in 300 mL Erlenmeyer flasks containing 100 mL WSP30 medium at 28°C, 120 rpm in either darkness for 4 days or under a 16 to 8 hour diurnal rhythm.

### Downstream

#### Cell disruption and solvent extraction of 1 mL cultures

The mycelia were transferred into 2 mL tubes. The cell disruption was performed directly in the solvent, ethyl acetate, using a SpeedMill Plus (Analytica Jena). Two different types of steel beads (RB-3 RS and RB-4 RS) and 1 mL ethyl acetate were added to the mycelium. A three minute disruption step at a frequency of 50 Hz was performed twice. Afterwards, the samples were centrifuged at 12000 rpm for 5 min (Allegra X-22R, Beckman Coulter) and 900 µL of the supernatant were transferred into a new 1.5 mL tube and evaporated to dryness. The extract was used for LC-MS-analysis.

#### State of the art extraction at 100 mL scale

After 4 days of cultivation the cultures were homogenized using an Ultra-Turrax at 16000 rpm (T25 basic IKA Werke, Germany) until complete homogenization was obtained (after approximately 20 seconds). 100 mL of ethyl acetate were added and a second homogenization for approximately 20 seconds was performed. The mixture was transferred to a separation funnel. The solvent phase was given into a round bottom flask. The evaporation of the solvent was carried out using a rotary evaporator. The dry extracts were then resolved in 1 mL of methanol and filtered through a 0.2 µm PTFE filter (Rotilabo-syringe filters, ROTH) via a syringe.

#### Adaptation of 100 mL extraction using PreCelly homogenization

After 4 days of cultivation (culture conditions see reference cultivation), culture broth was vacuum filtrated using glass microfiber filters (1.6 µm, GFA, Whatman). Approximately 0.5 cm^2^ of mycelium were transferred to a 2 mL lysis tube containing beads (ceramic beads, diameter of 0.4 to 0.6 mm, obtained from Analytic Jena). After addition of 1 mL ethyl acetate, two extractions for 45 seconds with delay of 20 seconds were performed using a PreCellys 24 device (Bertin Technologies) at 6500 rpm. After cell disruption, the samples were centrifuged for 10 min at 13000 rpm followed by a transfer of the solvent phase to a 1.5 mL tube. The solvent was evaporated employing a vacuum centrifuge. Biomass of the extracted mycelium was determined after subsequent drying of the remainings at 60°C.

#### Spiking for quantitative extraction control

To determine the quantitative success of the adapted extraction procedure, a spiking was performed by the addition of purified scopularide A in three different concentrations (0.1, 0.2 and 0.4 mg mL^−1^). 200 µL of the solutions from each concentration were added to the samples before extracting the compounds (n = 2). For comparison, samples from the same cultures were extracted without the addition of scopularide A.

### Analysis

#### LC-MS analysis for screening purposes

Extracts were dissolved in 250 µL methanol for extracts derived from 1 mL cultivation, and in 500 µL for extracts from 100 mL cultivation and prepared for LC-MS by using an ultra-sonic bath for 2 min, followed by a centrifugation step at 13000 rpm for 20 seconds at room temperature. The upper layers of the phases were transferred to a HPLC vial. 20 µL of the extract obtained from the 1 mL cultivation and 10 µL from the 100 mL cultivation were injected to a Hitachi Elite LaChrom system using a C18 column (Phenomenex Onyx Monolithic C18, 100×3.00 mm) applying a water (A)/acetonitril (B)/methanol (C) gradient (0 min: 5% B, 5% C; 1.5 min: 60% B; 4 min: 90% B; 4.5 min: 100% B; flow 2 mL min^−1^) with 0.1% formic acid added to A and B. The gradient was optimized for the detection of the two scopularides. The LC system was coupled to a benchtop time-of-flight spectrometer (mircOTOF II, Bruker Daltonics), calibrated with sodium formiate clusters solution (5 mM sodium hydroxide and water: 2-propanol 1∶1 (v/v) with 0.2% of formic acid). Electrospray ionization was optimized for a molecule size of *m/z* 600–750. QuantAnalysis Version 2.0, Bruker Daltonics GmbH was used for quantification.

The standardized LC-MS method protocol was applied as described by Silber et al. (2013) [14]. Manual analyses of data were performed using DataAnalysis Version 3.3, Bruker Daltonics GmbH.

#### Statistical calculation

For validation purposes, several statistical analyses were performed using arithmetical mean 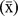, median (m), standard deviation (s) and coefficient of variation (V). The relative error (f) was specified with,
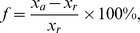
with the measured value 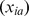 and the value 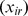 as determined by the linear regression (I) and (II) of the calibration curve of scopularide A (see section below). The number of replicates (n) is given for all experiments.

#### Linear regression for determination of the amount of scopularide A

Linear regression was applied to the linear correlating part of the calibration curve of scopularide A. In addition, the stability index was determined. Two linear regressions were performed with (I) and without (II) a calibration through the point of origin:
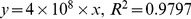
(I)

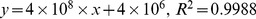
(II)For the determination of the amount of scopularide A in the sample, the linear regression (II) containing a background noise was applied. Equation (II) was used, solved for x:



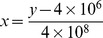



**Figure 2:**
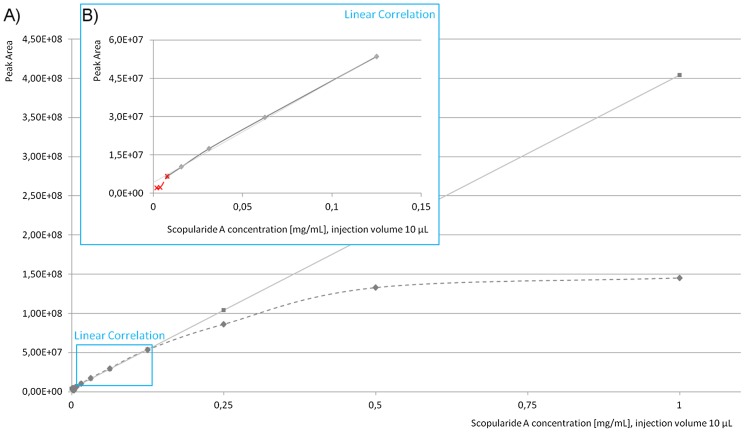
Calibration curve of scopularide A (ScoA) performed with micrOTOF II, Bruker Daltonics; a) Comparison of measured and theoretical peak area of scopularide A concentrations: (− −♦− − (dark grey)) measured peak area (injection volume 10 µL), (–×– (light grey)) theoretical peak area (based on linear regression I); b) Linear part of the graph: (–♦– (dark grey)) measured peak area, (− −×− − (red)) measured peak area, below the linear correlation (injection volume 10 µL), (–– (light grey)) linear regression y = 4×10^8^× x + 4×10^6^, R^2^ = 0.9988 background noise of 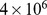. Linear area for calibration purposes was set to a range of 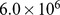 to 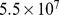 (peak area), equivalent to a scopularide A concentration of 0.008 to 0.125 mg mL^−1^.

The correlation of the concentration [mg mL^−1^] of scopularide A (x: abscissas) was determined by the peak area (y: ordinate) of the MS measurements (Figure 2a). For the evaluation of the theoretical peak area, linear regression (I) was used.

## Results and Discussion

**Figure 3:**
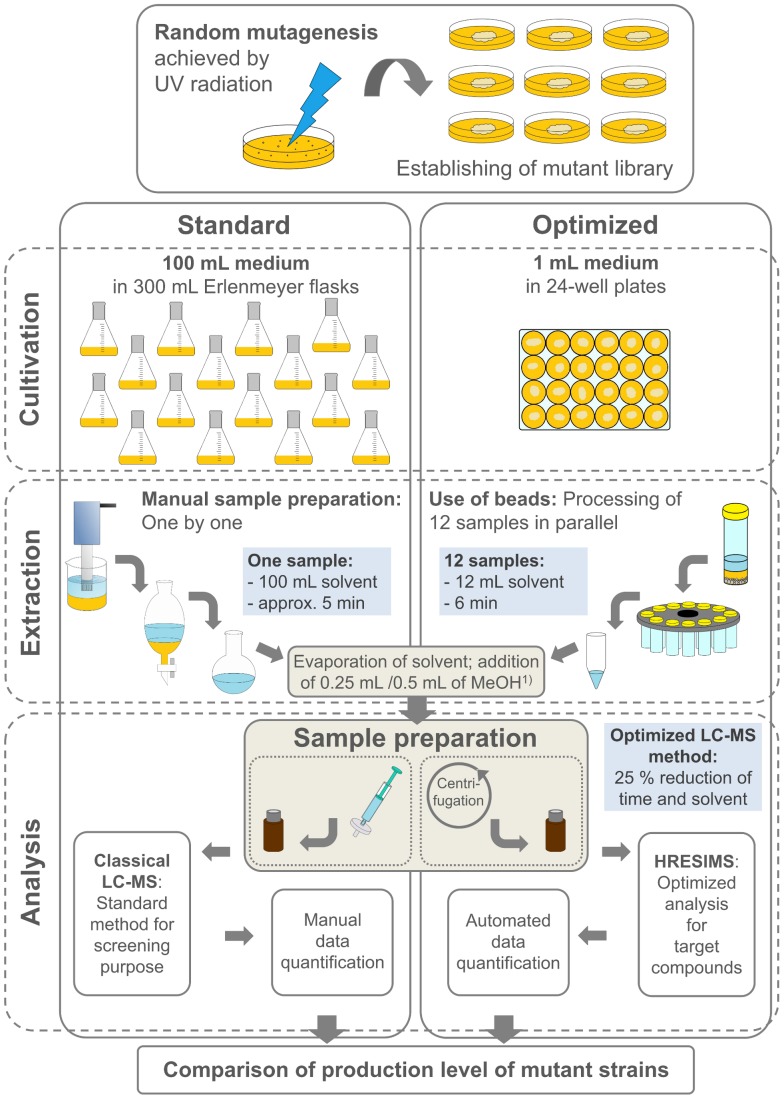
Comparison of standardized and optimized method: The miniaturization and optimization of the screening procedure was set up in three parts, starting with the initial establishing of the UV-mutant library. The cultivation in 24-well plates (upstream), the extraction (downstream) and the quantitative analysis including the sample preparation for LC-MS of scopularide A were compared separately to the standardized screening procedure in 300 mL Erlenmeyer flasks containing 100 mL medium.

An optimized quantitative and bioassay-independent screening system for the enhanced production of intracellular secondary metabolites was developed. The screening system was applied to characterize UV mutants of a marine *Scopulariopsis brevicaulis* strain (LF580) producing the anti-cancer active compounds scopularides A and B. The focus of the study was set on the amount of scopularide A, being the main product. Production of scopularide A was approximately 10-fold higher as production of scopularide B. The miniaturization and optimization of the screening procedure was set up in three parts: the cultivation in 24-well plates (upstream), the extraction (downstream) and the quantitative analysis including the sample preparation for LC-MS (Figure 3) of scopularide A. All parts were compared to a standardized screening procedure of LF580 cultures in 100 mL medium in Erlenmeyer flasks. Conclusions were drawn regarding time, cost and effort needed for quantification.

### Upstream part

#### Miniaturization of cultivation

The wild type strain of *Scopulariopsis brevicaulis* LF580 yielded an average dry biomass of 5.7 mg mL^−1^ ± 0.9 mg mL^−1^ (n = 11, V = 15.8%) when using the standardized cultivation conditions in 300 mL Erlenmeyer flask containing 100 mL medium. Miniaturization of the cultivation volume by a factor of 100 did not show a significant reduction or increase in cultivation time. A reproducible growth could be observed for the wild type in 1 mL medium in 24-well plates with an average dry biomass production of 9.4 mg mL^−1^ ± 3.3 mg mL^−1^ (n = 22, V = 35.1%) after 4 days of cultivation. Higher biomass values for small-scale cultivation processes were observed by Minas et al. 2000 for filamentous *Streptomyces* strains and were explained by the higher surface-to-volume ratio [15]. The advantage of the use of multi-titer plates is obvious, as they are recognized to be inexpensive, highly standardized and easy to handle [16]. The application of multi-titer plates for screening of mutant libraries of bacteria strains as well as yeasts and the related secondary metabolites has become standard practice in recent years [17]. There are only few reports on cultivation of filamentous fungi in 24-well plates [9], [10]. One main problem of small scale cultivation is a sufficient aeration. Using 24-well plate with a diameter of 16 mm of the wells does not implicate a significant reduction in aeration [18] and was thereby used in the recent studies using filamentous fungi [9], [10]. Therefore, a production of both scopularides was postulated and could be proven by LC MS. Beside a 100-fold reduction of medium for one strain, the space needed for cultivation was also decreased immensely, as a stacking of the multi-titer plates is technically given.

#### Influence of two different light-cycles

Furthermore, the influence of two different light-cycles was investigated, as it is known that light may have an impact on growth, as well as on the metabolite production of filamentous fungi [19], [20]. The Comparison of the growth of the wild type strain LF580 and the mutant strain M26 as well as the compound production made clear that light does not have an influence on both parameters. Biomass production represents a comparable value (6.2 mg mL^−1^ ± 1.3 mg mL^−1^ (n = 3, V = 21.5%)), the same holds true for the production level of the scopularides A and B, as well as the appearance of the phenotypes.

### Downstream part

#### Miniaturization of extraction

The extraction is the second fundamental step and was considerably reduced with respect to time, solvent consumption and waste as well as costs. Due to the small amount of material, a fast extraction procedure was performed, allowing to process up to 12 samples in parallel. This led to an approximately 10-fold reduction in time and 100-fold reduction in solvent requirement in comparison to the standard procedure performed at a 100 mL scale for each sample (see Figure 3). Hence, the reduction of solvent represents an enormous economical and ecological saving. In addition, the optimized method significantly reduces handling time, and results in a standardized extraction method enabling reliable comparison between the samples, as the manual extraction method showed a higher deviation from sample to sample, due to the fact that cultures were processed one by one with a high personal bias (time of homogenization, strength of shaking, etc.). The optimized extraction procedure was used in all subsequent determinations of the scopularides even at the 100 mL scale in order to reduce deviation and to ensure a comparability of the extraction grade from the different cultivation volumes. As a consequence, the sample dry weight was measured for normalization purposes.

#### Validation of miniaturized extraction

**Figure 4:**
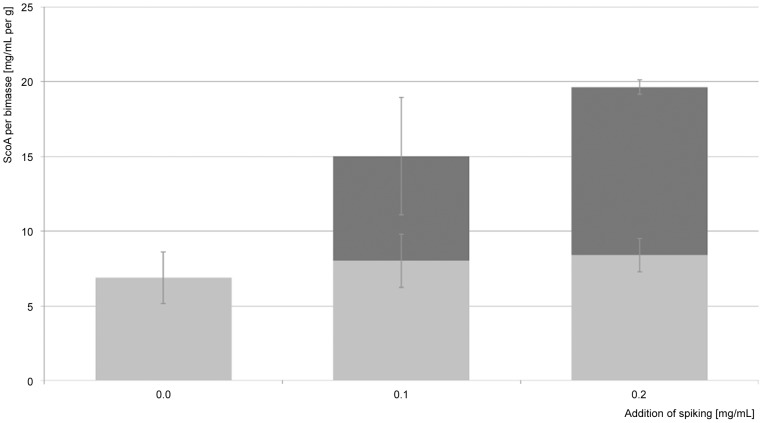
Spiking addition of scopularide A (ScoA), to determinate extraction comparability, results for 0.1 and 0.2 mg mL^−1^ are shown (scopularide A per biomass [mg mL^−1^ per g] of cultures plus spiking (dark grey) and scopularide A per biomass [mg mL^−1^ per g] of pure cultures (light grey)).

In order to determine the extraction efficacy of the adapted extraction method, a standard spiking experiment was conducted by adding scopularide A to mycelium of wild type samples (n = 2). The added concentrations of 0.1, 0.2 and 0.4 mg mL^−1^ scopularide A were detected by MS analysis (n = 4, V(0.1 mg mL^−1^) = 12.4%, V(0.2 mg mL^−1^) = 10.7%, V(0.4 mg mL^−1^) = 9.9%). A reliable extraction grade of the miniaturized extraction procedure on the basis of cell disruption using beads was assumed, due to the complete retrieval of the different added scopularide A amounts (Figure 4). The necessary inclusion of a cell disruption step is of disadvantage for this miniaturized screening approach as it includes additional complexity and effort. However, having the future need for upscaling for biotechnological purposes in mind, cell-bound metabolites are much easier to be captured as compared to released compounds, which have to be concentrated from high volumes.

### Analysis part

#### Optimization of the LC-MS analyses

To further reduce cost, filtration of each extract for LC-MS sample preparation was omitted. Instead extracts were centrifuged and the upper clarified phase was retained for LC-MS analysis. This step heavily reduces beside cost the time needed for sample preparation. By applying this method no significant pollution of the pre-column was observed. The adaptation of the LC-MS method to the target compounds led to a reduction of the LC run time of 2 min (25%) per sample. This was accompanied by a reduction of 25% of required solvents. To achieve higher sensitivity at lower compound concentrations a high-resolution mass spectrometer device was used for quantitative measurements.

#### Quantification of target molecules

**Figure 5:**
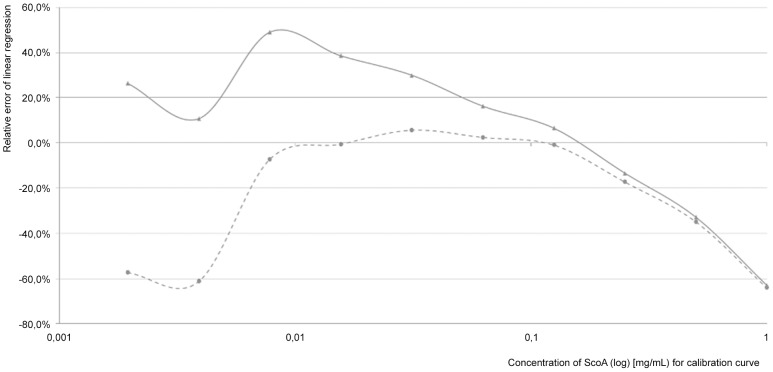
Comparison of relative error of measured values of both linear regression I (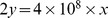: − −•− − (light grey)) and II (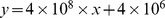: –▴– (dark grey)) in dependence of scopularide A (ScoA) concentration [mg mL^−1^] given for the measured values of the calibration curve (injection volume 10 µL). Linear regression II containing a background of 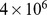 showed a much more defined agreement of the measured and calculated values within the linear area, as a distinct plateau was visible. Therefore a background noise was accepted for the calculation of the scopularide A concentrations.

Validation of quantitative screening methods included the approximation the origin of the occurring deviations, which define the accuracy and reliability of the method. Deviation between the samples can occur in each part of the process. For the determination of production level of scopularide A, the nature of deviation is totally different between the respective process steps used: cultivation, extraction and analysis. The main reason for deviation during cultivation was based on biological parameters, which must be differentiated into external and internal. In the design of our experiment, influence is only given on external parameters, like cultivation temperature and volume, shaking speed etc. However, the change of internal parameters (e.g. anabolism, metabolism) can be understood as an adaptation to the external ones. In contrast to the other parts of the method, the cultivation of a biological system (i.e. the fungus) represents a heterogenic and multi-component system, which is generally hard to predict. Therefore, the cultivation represented the part of the process with the highest system immanent deviation. Hence, a methodological change has the lowest influence on the reliability of the upscaling part. For the extraction part, a low technical deviation was achieved by the newly developed methodological set-up. The analysis as the third and final part of the process introduced variance through measurement and analysis. Therefore, this process part offered several options to enhance the reliability in the quantification of the metabolites in each sample. To optimize the accuracy within the measurements, a determination of the linear measuring range of the analysis was required. The subsequent analysis enabled multiple possibilities to enhance the accuracy, as several parameters can be adopted. Therefore, an additional focus was set on this part of the method. For the standard method, analysis of each sample was performed by manual peak area integration. As a consequence, the deviation was depended on a personal influence. For the optimized quantification the software QuantAnalysis was used. This software tool allowed the automated quantification of samples based on the integration of the peak area of a specific *m/z* value. In addition, the sensitivity of the MS-dependent quantification was enhanced by optimizing the ionization process for molecules in the range of *m/z* 600 to 750. Two *m/z* values were used for quantification: the positively charged *m/z* value of scopularide A [M+H]^+^ and the *m/z* value of the respective sodium adduct [M+Na]^+^, which was also generated during the process of electrospray ionization as sodium was present in the system. The quantification was based on both values as the ratio of the pseudo-molecular-ions varied from run to run. Furthermore no additional *m/z* values related to scopularide A were visible in the analysis. The samples were not desalted, since the calibration of the MS system was performed with sodium formate. This calibration is needed to minimize the technical deviation. Furthermore, an additional step in sample preparation was not desired. Applying the quantification with both *m/z* values, the accuracy of the LC-MS analysis could be optimized to a variation of 5% (given as (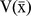) with n = 11) within the linear correlation range. Using this newly developed approach, a reduction of the deviation from 10–15% to 5% could be achieved. The use of a quantitative software tool facilitated the analysis of several samples in parallel, whereas the manual analysis had to be performed sample by sample. In addition, the measurement of the samples by a high-resolution mass spectrometer led to higher sensitivity and was thus adopted for the analysis. To ensure the linearity of the ionization and to end up with a concentration range required for a quantitative analysis, a calibration curve of scopularide A was obtained (Figure 2a). This curve showed a distinct linear correlation for the ionization and therefore, defined the range for quantitative measurements to a signal intensity of 6.0×10^6^ to 5.5×10^7^ (peak area), which is equivalent to a concentration range of 0.008 to 0.125 mg mL^−1^ of scopularide A (Figure 2b). The linear correlation was given with a stability index of R^2^ = 0.9988, setting the background noise to 4.0×10^6^. The acceptance of a background noise of 4.0×10^6^ led to an increase of the stability index of 2% from R^2^ = 0.9797 to R^2^ = 0.9988. A correlation between the intersection of 0 mg mL^−1^ scopularide A and the related peak area of 0 with the stability index of R^2^ = 0.9797 is only slightly less than the preferred value of R^2^ = 0.9988. However, a comparison of the relative error of both linear regression curves indicated that the linear regression containing a background noise of 4.0×10^6^ has a much more defined agreement of the measured and calculated values within the linear area, as a distinct plateau was visible (Figure 5). In contrast, a clear downtrend for the complete concentration range was observed when calculating the relative error without any background noise. The acceptance of background noise to the calibration model raised the sensitivity in the lower concentration range. Nevertheless, scopularide A concentrations below 0.008 mg mL^−1^ (Figure 2b) were not detectable, due to the accepted background noise of 4.0×10^6^ (peak area).

#### Application of the optimized screening process for the evaluation of UV mutants of *Scopulariopsis brevicaulis* LF580

The optimized screening system was used to screen mutants of *Scopulariopsis brevicaulis* LF580 with respect to compound production. A total number of 640 mutant strains were obtained by application of UV radiation in a two-stage approach. Initially, the wild type strain LF580 was exposed to UV light, resulting in 220 mutants. One of the mutant strains, M26, showed higher levels of scopularide A production. Besides, M26 had a changed phenotype. Subsequent characterization showed a shorter exponential growth phase, resulting in higher biomass rates per time, combined with a faster production of the target compounds. Subsequently, this mutant strain was irradiated leading to 400 second-generation mutants. All of these mutant strains were cultivated and screened using the optimized method. The focus of the assessment was set on the upstream part, since the downstream and analysis part of the optimized method were already validated via purified scopularide A and extracts derived from 100 mL cultures of the wild type strain.

#### Growth behavior of *Scopulariopsis brevicaulis* LF580 and selected mutant strains in 1 mL

24 strains, including the wild type strain and the mutant strain M26, were chosen for a distinct comparison of the optimized method and the standard approach. All strains showed decent growth using described conditions, and production of the target compound scopularide A was observed. Compared to the 100 mL cultivation conditions, the wild type strain did not show growth in pellets within the 24-well plate format, but formed agglomerated mycelia. This morphological characterization in 1 mL of liquid culture was also observed for the mutant strains. During 100 mL cultivation some mutant strains did not show such a clearly pellet shaped formation compared to the wild type. Besides biomass and scopularide production, the change of phenotype was a third criterion for the characterization of the mutant strains.

#### Proof of concept of miniaturization of cultivation

Strain LF580 represents a fungal secondary metabolite producer with a stable production profile on qualitative level, i.e. the two main metabolites scopularide A and B were produced under all various conditions used in this study. However, the amount of the metabolites varied: Deviations within the replicates (n = 3) in 1 mL and 100 mL were visible. In order to distinguish between measuring and technical errors as well as biological deviation, a comparison of all parts of the method was performed: As shown above, the deviation caused by technical and measuring aspects during extraction was reduced to 10–15% and in the analysis to 5%, respectively (given as coefficient of variation). This refers to the common experience that parallel execution of experiments with reduced manual intervention increases reliability [21]. For the downstream part of the procedure, a similar reduction was obtained by avoiding the two weak points of the standardized method: the manual extraction and the sample preparation. These were replaced by a standardized extraction method, sample preparation and quantitative analysis. In conclusion, the biological features cause the differences seen between the variability in scopularide production determined for the two cultivation scales. For the 100 mL screening approach, an overall mean deviation of 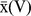 = 22% was observed. The variation coefficient in relation to the median (m(V) = 16.4%) was somewhat smaller. As shown in table 1, two third of the samples lay beneath the mean deviation. In contrast, the 1 mL cultivation showed a three-time higher mean deviation and a median of the coefficient of variation (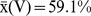, (m(V) = 62.3%) (table 1). This deviation in the cultivation part was likely due to biological features, i.e. metabolic deviation found between cells of the same strain. This observation is in contrast to the description of citric acid production using *Aspergillus carbonarius,* having a two times lower coefficient of variation for small scale cultivation (24-well plates) compared to Erlenmeyer flasks [9]. Such differences between two fungal species may occur in the fungal mycelia, which can be heterokaryotic in nature or show distinct growth behavior at all. Hence, *Scopulariopsis brevicaulis* LF580 - a non-model organism - does exhibit higher variation in small scale cultivation. Influences of deviation on the level of single cells in a cultivation system are higher in smaller cultivation volumes compared to larger volumes. This is due to the fact that upscaling can be regarded as an increase in the number of replicates, resulting in a higher number of single cells in the culture volume. Nevertheless, performing a higher number of replicates in small-scale cultivation decreases the deviation. The use of a higher number of replicates has the additional benefit that instable mutant strains can be pointed out earlier in the screening procedure. Small-scale fermentation represents the preferred tool for screening applications, as increasing the number of replicates does not proportionally increase process time, cost and effort [22]. Therefore, miniaturized screening processes open the possibility of performing parallel experiments, and give the opportunity to exploit large mutant libraries [23]. The small-scale cultivation process has still a weakness to overcome, as the biological systems of microorganisms have a higher variability compared to technical or chemical systems. The interest of designing miniaturized screening methods is not restricted to mutant libraries. The quick and easy development of cultivation processes are of interest for application [24] and functional screenings for the analysis of e.g. microbial lifecycles [25].

**Table 1:** Comparison of arithmetical mean and median of scopularide A production for 1

	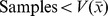	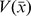	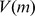
100 mLcultivation1)	**60.8%**	22.2%	**>**	16.4%
1 mLcultivation2)	**33.3%**	59.1%	**<**	62.3%

The coefficient of variation (V) is given for the arithmetical mean 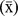 and the median (m) for scopularide A production (n = 3) in 100 mL and 1 mL cultivation format of 22 randomly selected mutant strains, the wild type strain LF580 as well as the mutant strain M26. In addition, the percentage of samples beneath the arithmetical mean is given in bold numbers. Two third of the samples of the 100 mL cultivation laid beneath the mean deviation. In contrast, the 1 mL cultivation showed a three-time higher mean deviation and a median of the coefficient of variation.

1) 23 respectively.

2) 21 samples were evaluated.

## Conclusion

Biological variation is an issue for all screening technologies available; however sufficient information on the influence of biological in contrast to methodological variation is scarce. Hence, our main issue was the development of a robust and reliable screening system applicable generally for the search of secondary metabolites in fungi. A mutagenesis experiment was chosen as a respective application example.

The optimized screening procedure was evaluated and validated in three parts: the cultivation (upstream), the extraction (downstream) and the analysis, including sample preparation for LC-MS (Figure 3). The focus during the validation of the miniaturized screening method was set on the amount of scopularide A. For optimization the cultivation was downscaled from 300 mL Erlenmeyer flask to 24-well plates. Importantly, the change of cultivation conditions did not show any qualitative influence on the metabolite production. An optimization of the extraction procedure was performed based on the reduced cultivation volume combined with a smaller sample size. A small-scale extraction procedure was established with a metabolite yield sufficient for LC-MS analysis. The preparation of the samples for LC-MS analysis was optimized in effort and cost without having any observable negative influence on the subsequent analyses. The modification of the LC-MS method led to an additional reduction in time and cost.

Setting the focus on the downstream and the analysis part of the method, we could establish a reliable process. The miniaturized upstream process has still a weakness to overcome, as a higher variation was observed in the miniaturized system, which can be explained by biological parameters. However, biological variation is an issue for all screening technologies.

In our manuscript, we show for the first time, that by a coupling of miniaturized methods in both, fermentation and downstream, a direct, bioactivity-independent quantification of secondary metabolites is possible.

As a proof of concept, we showed the application of the optimized method for the screening of UV mutants of *Scopulariopsis brevicaulis* LF580 with regard to an enhanced production of the target secondary metabolites. The analyses underlined how the separate validation and statistical consideration of each step of the process can help to distinguished between the origins of the deviation in order to minimize them. The developed method led beside an enhanced reliability, to a remarkable reduction of work time. However, quantification should be performed for the upstream part for each strain individually in order to consider and limit strain specific biological variation. The optimized screening procedure developed for mutant selection of *Scopulariopsis brevicaulis* LF580 can easily be adopted for similar purposes with other filamentous fungi due to its activity-independent screening approach.
